# 

**Published:** 1860-08

**Authors:** 


### CONTENTS.

ORIGINAL ESSAYS AND COMMUNICATIONS.

Selection and Arrangement of Teeth, in constructing Dental Substitutes. By PROF. W. CALVERT, D. D. S. .......... 65
"Fang Filling." By A A BLOUNT, M. D. .......... 70
Filling Teeth. By DR. H. A. SMITH .......... 75
Treatment of a Diseased Tooth. By J. T .......... 80
Fang Filling, and Treatment of the Alveolar Abscess. By DR B. A. ROSE .......... 81
Compatability. By J. T .......... 82

PROCEEDINGS OF SOCIETIES.

Cincinnati Dental Association .......... 85
Mad River Valley Dental Society .......... 89
Proceedings of the Western Dental Association .......... 91

CORRESPONDENCE .......... 94

SELECTIONS .......... 97

EDITORIAL .......... 117

